# *Mycobacterium avium* Subspecies *paratuberculosis* Infection in Zoo Animals: A Review of Susceptibility and Disease Process

**DOI:** 10.3389/fvets.2020.572724

**Published:** 2020-12-23

**Authors:** Marco Roller, Sören Hansen, Tobias Knauf-Witzens, Walter M. R. Oelemann, Claus-Peter Czerny, Ahmed Abd El Wahed, Ralph Goethe

**Affiliations:** ^1^Zoological-Botanical Gardens Wilhelma, Stuttgart, Germany; ^2^Department of Animal Sciences, Faculty of Agricultural Science, Institute of Veterinary Medicine, Division of Microbiology and Animal Hygiene, Georg-August-University Göttingen, Göttingen, Germany; ^3^Institute for Microbiology, University of Veterinary Medicine Hannover Foundation, Hannover, Germany; ^4^Department of Immunology, Institute of Microbiology, Federal University of Rio de Janeiro, Rio de Janeiro, Brazil

**Keywords:** *Mycobacterium avium* subspecies *paratuberculosis*, MAP, paratuberculosis, Johne's disease, zoo animals, ruminats, non-ruminants

## Abstract

*Mycobacterium avium* subspecies *paratuberculosis* (MAP) is the causative agent of paratuberculosis (ParaTB or Johne's disease), a contagious, chronic and typically fatal enteric disease of domestic and non-domestic ruminants. Clinically affected animals present wasting and emaciation. However, MAP can also infect non-ruminant animal species with less specific signs. Zoological gardens harbor various populations of diverse animal species, which are managed on limited space at higher than natural densities. Hence, they are predisposed to endemic trans-species pathogen distribution. Information about the incidence and prevalence of MAP infections in zoological gardens and the resulting potential threat to exotic and endangered species are rare. Due to unclear pathogenesis, chronicity of disease as well as the unknown cross-species accuracy of diagnostic tests, diagnosis and surveillance of MAP and ParaTB is challenging. Differentiation between uninfected shedders of ingested bacteria; subclinically infected individuals; and preclinically diseased animals, which may subsequently develop clinical signs after long incubation periods, is crucial for the interpretation of positive test results in animals and the resulting consequences in their management. This review summarizes published data from the current literature on occurrence of MAP infection and disease in susceptible and affected zoo animal species as well as the applied diagnostic methods and measures. Clinical signs indicative for ParaTB, pathological findings and reports on detection, transmission and epidemiology in zoo animals are included. Furthermore, case reports were re-evaluated for incorporation into accepted consistent terminologies and case definitions.

## Introduction

Mycobacterial infections in zoo animals can be of significant relevance in terms of animal welfare and conservation efforts. Diagnosis and management strategies need to consider the risk of transmission of *Mycobacteria* from infected or suspicious animals to the zoological collection, as well as the potential zoonotic hazard of the pathogens.

This review focuses on the occurrence and epidemiology of *Mycobacterium avium* subspecies *paratuberculosis* (MAP) in animals managed in zoological gardens. The susceptibility to MAP of free-ranging and farmed wildlife is only partially addressed as it has already been extensively reviewed (1–5).

Exotic species housed in a zoo environment face epidemiological situations similar to those in livestock herds (e.g., high animal density and exposure to high concentration of infectious agents in the population). This may lead to an increased infection pressure and population stress compared to free-ranging animals, where paratuberculosis (ParaTB) does not appear to be extensive on herd level nor geographically widely distributed (6). However, results of a recent review by Whittington et al. [(7); Supplementary Table 5. Free living wildlife species with MAP infection] showed MAP infection in wildlife in 18 (38%) of 48 examined countries while in 26 countries the situation was unknown. Infection in wildlife may therefore be much more extensive and geographically widespread than we already understood.

Several publications and review articles comment on diagnosis, prevention, and control of ParaTB in zoological gardens, where the disease threatened the valuable animal collections of exotic and endangered species. To date, systematic surveys on MAP infection in zoo animals are unavailable for many species and most studies are limited to various ruminant species. In addition, differences in diagnostic methods together with limited final pathogen confirmation make it difficult to compare these reports.

The aim of this review is to re-evaluate recent literature on susceptible and affected zoo animal species and taxonomic groups considering applied diagnostic approaches and varying case definitions. Whenever possible, the reports were incorporated into defined case definitions according to Whittington et al. (8). Thereby, the implementation of conceptual ranking of evidence for case definition enables the classification of individual animals or herds in terms of pathogenesis and allows illustrating susceptible families.

### ParaTB: General Remarks

The etiological agent of ParaTB, a chronic and slowly progressive granulomatous enteritis of small and large domestic ruminants, is *Mycobacterium avium* subspecies *paratuberculosis* (9). MAP is a small, acid-fast, rod-shaped, aerobic, and facultative intracellular bacterium of the *Mycobacterium avium* complex (10). ParaTB is reportable in some countries, occurs worldwide, and progressively spreads in global livestock industry, leading to significant economic losses and considerable impact on animal husbandry and welfare (11, 12).

### Epidemiology, Host Range, and Susceptibility

Clinical ParaTB has been diagnosed in a wide diversity of free-ranging and captive exotic artiodactyls (13–15). However, MAP infections of non-ruminants such as odd-toed ungulates, lagomorphs, rodents, macropods, carnivores, non-human primates and birds have also been reported (6, 16).

MAP is classified into two major strain types; type S (Sheep type with subtypes I and III) and type C (Cattle type or Type II; including type B: USA and Indian Bison Type). Type S strains are predominantly found in sheep and goats but are uncommon in wildlife (17). In contrast, the common type B strain in cattle has a broad host range, including both ruminants and non-ruminants (2). Cross-species infection and sharing of specific strains between wild and domesticated animals have been shown in several studies (18, 19).

### Pathogenesis, Transmission, and Zoonotic Potential of MAP

Characteristics of MAP infection and disease depend on the host species and are best known for ruminants. Whitlock and Buergelt (20) defined four stages for ruminant ParaTB; STAGE I: Silent infection of calves, young livestock and adults; STAGE II: Subclinical disease of carrier adults; STAGE III: Clinical disease; STAGE IV: Advanced clinical disease in few animals. Infection is commonly latent and asymptomatic. Shedding animals in stages II and III spread the pathogen intermittently or chronically and represent an often-unrecognized reservoir for MAP. Therefore, these animals are of major epidemiological significance (21, 22). Co-housed individuals and offspring are at highest risk of infection (23, 24), which occurs after fecal-oral pathogen contact, mostly during the first weeks to months of life (25). Transmission to a susceptible livestock host occurs mainly by vertical infection in neonates or animals in the postnatal period, either through sucking the manure-contaminated teats or, later on, by the uptake of feed contaminated with feces (26). Vertical and pseudo-vertical transmission from clinically diseased and infectious dam (e.g., *in utero* infection), or by colostrum and milk has been described (27–29), and the possibility of venereal transmission by semen from domestic bulls was reported (30).

Establishment and course of infection depend on the amount of ingested pathogen, the route of infection as well as age, immune status and physical and genetic resistance of the affected animal. Furthermore, bacterial and environmental factors, strain variations and a variety of other stressors seem to be involved (31). It is largely accepted that M-cells of the Peyer's patches in the ileum mediate MAP uptake from the intestinal lumen. Once in the subepithelial mucosa, MAP is engulfed by intestinal phagocytic cells. Bacteria are able to grow in phagocytes and can disseminate within the jejunal and ileal mucosa and spread to regional lymph nodes (32). Infected animals develop an initial Th1 cell-mediated immune response, which might control bacterial spread and results in either bacterial clearance or subclinical infection. However, stress and other unknown triggers in the chronic phase lead to a Th2 humoral immune response, which fails to contain the infection (33).

Whether MAP can be regarded as a potential public health issue and pathogenic in humans is inconclusive and cannot be definitively answered (34, 35). Higher prevalence of MAP in humans with Crohn's disease suggest a zoonotic risk (36). A causative relationship is still not confirmed but should be considered in discussions about hygiene concepts for “petting zoos” and long-term exposure of zoo animal keepers.

### Clinical Signs of ParaTB

Clinical signs of ParaTB, primarily observed in adults, can considerably differ among different ruminant species and are usually absent until advanced stages of the disease. The clinical manifestation usually follows situations of increased stress. Transportation, malnutrition, overcrowding, parasitic infestations, mineral deficiencies, calving, lactation period or concentration and reorganization of animal groups may influence and enhance the disease course and represent contributing factors (37–39).

The bacteria appear to populate intestinal macrophages and wait for the best opportunity to multiply, spread and elude immunologic control (40). Subsequently, the pathogen multiplies in the macrophages and causes progressive granulomatous inflammation in the intestinal tissues and associated lymph nodes, and the amount of fecal shedding continues to increase.

Classic clinical ParaTB is characterized by an extended granulomatous and incurable enteritis with or without diarrhea, leading to wasting and gradual emaciation despite an uninfluenced feed uptake (9). While profuse diarrhea and intermandibular edema are usually characteristics of late stages in cattle, clinical signs in sheep and goats are limited to chronic weight loss, an unkempt appearance and deteriorated condition and lethargy. Softer feces or diarrhea are rarely seen and may only display in the terminal stages (41). Clinical signs of the disease are mostly inapparent, but once clinical manifestations are evident, the animal rapidly deteriorates and the disease is regularly fatal.

However, as stated above, the presentation of the disease in domestic and non-domestic ruminants as well as in other species can be markedly different (2).

### Pathology

Pathological findings are also often non-specific and can differ in affected individuals. In addition, not all species develop gross pathology (42). Gross *post-mortem* findings may include cachexia; atrophy of fat tissue; and macroscopic thickening, hyperemia, erosion, and corrugation of the intestinal mucosa, predominantly in the terminal ileum. Associated mesenterial and ileocecal lymph nodes and the ileocecal valve may be enlarged, edematous and the afferent lymphatic vessels are possibly blocked and corded (20). Caseation and calcification of lesions are rare but might occur in small ruminants, cervids, and the South American camelids (41). This leads to difficulties in distinguishing the lesion from tuberculosis and other mycobacterial diseases.

Histological lesions vary from mild to severe and paucibacillary to multibacillary and present histiocytic granulomatous inflammation which may cause diffuse mucosal thickening and atrophy of intestinal villi and glands, accompanied by decreased absorptive capacity and functional loss (25). Although acid-fast bacteria (AFB) typically reside in epithelioid macrophages and multinucleated giant cells forming nests in the intestinal mucosa and mesenteric lymph nodes, the infection can generalize in advanced stages (43) and granulomatous lesion may also be found in the distal jejunum, caecum and colon as well as in associated lymph nodes and other vital organs (e.g., liver, lung).

### Diagnosis

Diagnosis of ParaTB is influenced by the course of infection and immune response of the affected animal. Chronicity and low incidence of disease limit *ante-mortem* diagnosis, the classification of infected individuals, and the characterization at each stage (44). Intermittent excretion, heterogeneous distribution and potentially low numbers of MAP within a fecal sample reduce the significance of pathogen detection, which requires a repeated and regular sampling and testing to increase the probability of detecting shedding individuals. Course of disease, clinical signs in individual animals, unresponsiveness to treatment and acid-fast positive lesions during *post-mortem* examination allow reasonable diagnostic clues. However, in some cases, especially in wild and farmed deer, pathological lesions of ParaTB may not be distinguishable from lesions caused by *Mycobacterium bovis* and other *Mycobacterium avium* subspecies (4).

Various *ante-mortem* and *post-mortem* tests, either direct for pathogen detection, as well as indirect for humoral immune response can support or confirm the suspicion (45). All diagnostic methods available to date tend to underestimate true infection and disease prevalence and lack reliability, especially during early and preclinical stages. Variation between the results of different tests are common and false-positive or -negative test results reflect the difficult disease confirmation (46). For reliable and convincing *ante-mortem* diagnosis in individual animals, tests should always be combined with objective historical evidence and epidemiological disease assessment (8).

Cultivation of the pathogen from intestinal tissues is commonly used in diagnosis but should always be confirmed by PCR. Culture requires incubation for weeks to months on appropriate, mycobactin-supplemented culture media before small, fastidious, round and whitish colonies can be observed (47). The efficiency of MAP isolation differs between strain types. Type S strains typically grow more slowly and are more difficult to cultivate (48).

Molecular biology methods for diagnosis, species identification and typing offer a sensitive, specific and rapid detection with reduced diagnostic time and a potentially higher sensitivity (49). MAP is identified by amplification of specific DNA sequences such as IS900 (15–17 copies per MAP cell), HspX (one copy per MAP cell), ISMap02 (six copies per MAP cell), F57 (one copy per MAP cell) and the genomic locus 251. ISMav02 (3 copies per MAP cell) is another target that is used but no longer considered MAP-specific (50–52).

MAP-specific cell-mediated immune response can be detected by the intradermal skin test, using Johnin or avian purified protein derivative (PPD), or the gamma interferon assay (IFN-γ-test). The skin test was used more frequently in the past, but its current use is limited (53).

Antibody-detection-based tests include enzyme-linked immunosorbent assays (ELISA), agar gel immunodiffusion and complement fixation. Clinical specificity is usually high, while clinical sensitivity depends on the stage of ParaTB and tends to be low in the subclinical stage with lower bacterial load prior to the appearance of clinical signs (46). Commercial ELISAs intended for use in domestic ruminants are generally not validated for non-domestic animals and therefore may be either limited or not suitable to detect antibodies in these species. Hence, it is difficult to appraise critically results of many serological surveys that have previously been performed for MAP detection in wildlife (54). Vansnick et al. (55) used an indirect ELISA (HerdCheck *M. paratuberculosis* ELISA; IDEXX Laboratories, Inc., Westbrook, Maine; from here on referred to as HerdCheck ELISA) based on a non-species-specific binding conjugate (protein G) in zoo animals as an alternative to species-specific secondary antibodies. Obtained positive results might therefore indicate exposure to MAP. However, similar to pathogen detection assays negative results in immunodiagnostic tests are not reliable for diagnosis. They must be evaluated with caution considering availability, applicability and particularly the accuracy of the implemented method as measured by diagnostic sensitivity and specificity in different disease stages (44).

According to the classification of Whittington and coworkers [Table 1; (8)], which was also applied for the case definitions in this review, in terms of efficient prevention and control of ParaTB, it is essential to differentiate between exposed, infected and infectious animals, as well as between subclinically and clinically diseased individuals. If possible, detection of MAP should be incorporated in the definition of an identified case.

**Table 1:** Value of diagnostic findings; adapted from Whittington et al. (8).

**Findings**	**Diagnostic value**	**Limitation**
Clinical signs (Cs)	Suspicious	Non-specific, may be absent (depending on age, species, and the stage of the infection/disease)
Gross pathology (Gp)	Suspicious	Non-specific, may be absent (depending on age, species, and the stage of the infection/disease)
Histopathology (Hp)	Suspicious	Non-specific, may be absent (depending on age, species, and the stage of the infection/disease); (paucibacillary vs. multibacillary lesions)
Acid-fast bacteria (AFB)	Suspicious	Confirmation by PCR for appropriate genetic targets is needed because AFB other than MAP may be present
Culture of feces (C-f) (confirmed)	Exposed	Confirmation by PCR for appropriate genetic targets is needed Can provide indirect evidence of infection CAVE: Passive shedding (Pass-through) In the case of positive follow-up diagnostics suggestive of infection
Culture of tissue (C-t) (confirmed)	Infected	Confirmation by PCR for appropriate genetic targets is needed
Genome in feces (G-f)	Exposed	Can provide indirect evidence of infection CAVE: Passive shedding (Pass-through) In the case of positive follow-up diagnostics suggestive of infection
Genome in tissue (G-t)	Infected	
Serology (Se)	Suspicious	Alone not sufficient to define infection ELISA tests are not validated for most non-domesticated species False-positive immunological reactions are possible

Accordingly, animals are susceptible when they develop infection and disease (clinical or subclinical) after natural or experimental exposure to a sufficiently high number of infectious bacteria.

An animal is considered infected, if either culture or PCR from a tissue sample demonstrate a positive result. Culture or PCR from feces provide strong evidence of exposure but do not confirm infection, since a fecal “pass-through” phenomenon and therefore a passive shedding without infection may occur subsequently to an oral ingestion of MAP in a heavily contaminated environment. Thus, a positive fecal culture or PCR should be presumptive of ParaTB unless there is a history of multiple infected animals at the institution.

Direct fecal or tissue microscopy and ELISA-positive results are as well not sufficient to define infection. Positive results on more than one occasion increase the confidence about infection and may be suggestive and diagnostically persuasive.

The animal is considered diseased when histopathological lesions consistent with ParaTB are demonstrated and AFB in the lesions are confirmed to be MAP. Diseased individuals may come down with associated clinical signs or remain in a subclinical stage with no attributable clinical signs.

### Treatment and Vaccination

Treatment of ParaTB is usually not attempted or indicated because of the low likelihood of eliminating infection, leading to remission rather than cure, and the high costs of antimycobacterial drugs (56). Vaccination may reduce the risk of transmission, the number of clinical disease cases and the level of shedding (57). Implementation is limited as vaccinated animals interfere with serological testing for MAP as well as surveillance programs for tuberculosis due to the non-specific response to tuberculin skin tests. Both, vaccines and palliative treatment protocols might be considered for breeding purposes in individuals of exceptional genetic value or endangered species.

### MAP Infection and ParaTB in Ruminant and Non-ruminant Zoo Animals

Literature concerning zoo animals focuses almost exclusively on emergence of cases of suspicion or detection of MAP infection and ParaTB in zoos. Some studies also report on the measures initiated to reduce spread of MAP.

For the present review, literature search was realized using combinations of the key words “paratuberculosis” OR “Johne's disease” AND “zoo” in admitted scientific online databases [e.g., *via* VetSearch (University of Veterinary Medicine Hannover, Foundation) a combined literature search in PubMed, Web of Science, MEDLINE, CAB Abstracts, AGRIS, Academic OneFile, Base, SciELO, Wiley, ScienceDirect, SpringerLink, local catalogs]. Manual literature search techniques were employed to access reports of conference proceedings of the AAZV (American Association of Zoo Veterinarians) and EAZWV (European Association of Zoo and Wildlife Veterinarians) and their predecessor organizations. The last date on which the literature search was realized was December 31, 2019. Duplicative references were deleted.

**Figure 1:**
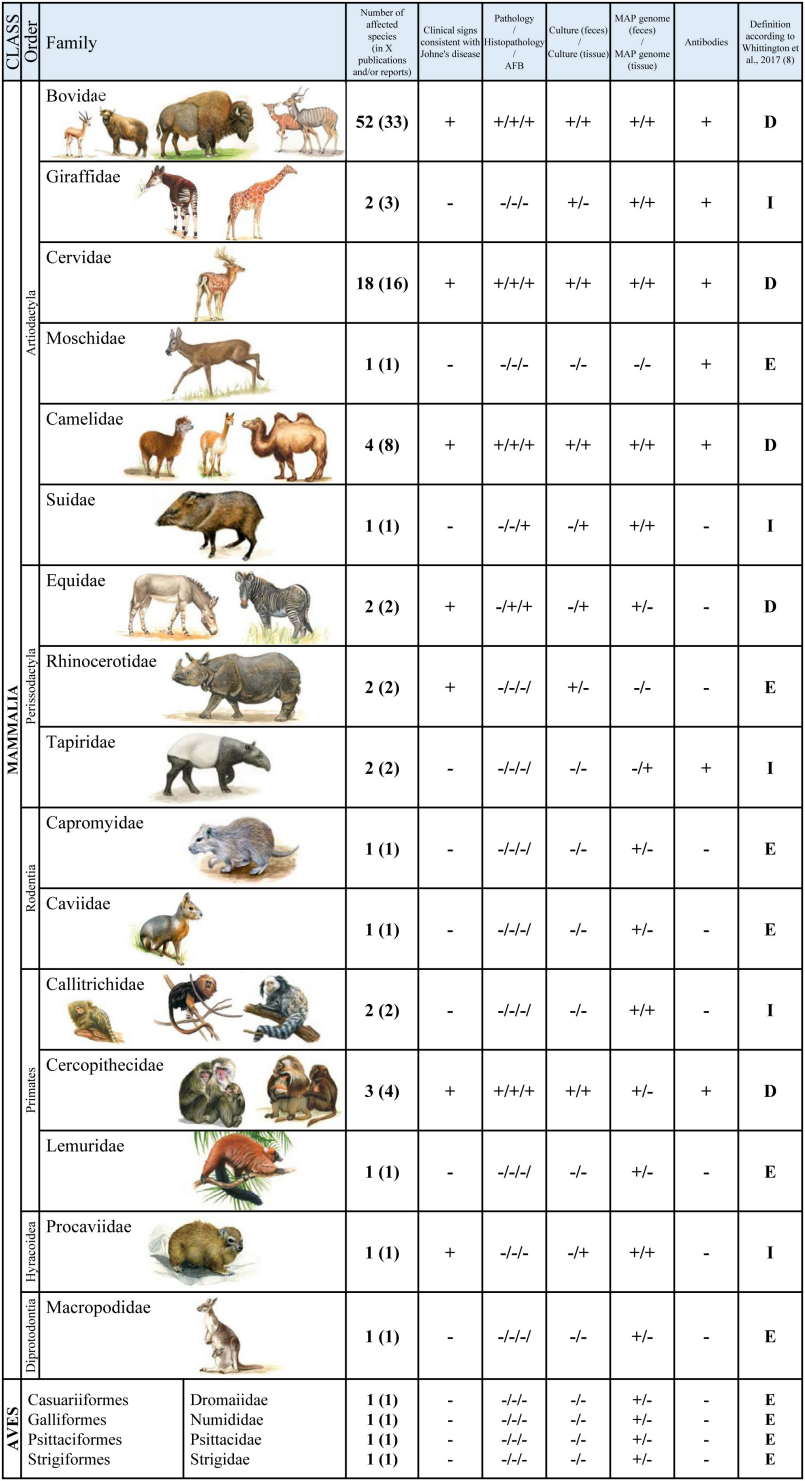
Summarized cases in zoo animals by order and family: “+”: described for/detected in the respective family, “-”: not described for/not detected in the respective family; “D” diseased, “I” infected, “E” exposed.

The presence of clinical signs, pathological and histopathological findings as well as associated diagnostics (serology, culture or molecular biology techniques using feces or tissue samples) were reevaluated according to Whittington et al. (8). Cases are summarized by order and family of the animal and presented in Figure 1.

A detailed and extensive register of the cases and their classification is listed in the Supplementary Table 1. This table can be used to access the diagnostic methods used for each individual report, provides a comprehensive overview of susceptible animals and, if possible, enables a classification according to the case definitions mentioned.

### Ruminant Zoo Animals

Ruminants represent the largest group of zoo animals identified with MAP infection and ParaTB. Like in studies on free-ranging animals, infection and clinical disease in zoos is described primarily in bovids and cervids, which represent the two predominant artiodactyl families kept in zoos. A limited number of reports describe the detection of MAP in giraffidae and moschidae. No reports for other taxonomic groups of ruminants (antilocapridae and tragulidae) were found either in managed care or in free-ranging populations.

#### Artiodactyla

##### Bovidae

Clinical signs and pathological lesions in non-domestic bovids correspond to descriptions in domesticated species and have been documented for many genera. As mentioned above, differences in the appearance of ParaTB in cattle, sheep and goats are possibly related to infection by different strain types. Such differences are also found in exotic species of this family, but nevertheless one must acknowledge that data are incomplete to define conclusive species-specific differences.

ParaTB in captive wild animals was firstly reviewed by Katic (58). Jármai (59) described evidence of ParaTB in a common wildebeest (*Connochaetes taurinus albojubatus*) with chronic diarrhea, hypertrophic enteritis, as well as AFB and caseation in mesenteric lymph nodes and liver. Dobberstein (60) reported the disease in an African buffalo (*Syncerus caffer*). Subsequently, early reports from German zoos also included clinical cases with typical lesions in domesticated West African dwarf goats (61). In 1972, Brahm et al. (62) described a granulomatous enteritis and lymphadenitis with extra- and intracytoplasmic acid-fast organisms and a remarkable involvement of spleen and liver, but minor gross lesions in the intestines, in enzootic cases in a large herd of blackbuck (*Antilope cervicapra*) in a German zoo. Such mycobacterial dissemination has been described before by Pallaske (43) in one animal of the same group, indicating that this type of propagation is not uncommon for blackbuck. Clinical signs in this species included severe persistent diarrhea, restricted motion, emaciation and a rapid decline in clinical condition.

ParaTB was histologically confirmed in smaller zoo ruminant species [Nubian ibex (*Capra nubiana*), bezoar goat (*Capra aegagrus*), blackbuck and cervids] in Germany, where the disease appeared to be frequent and the contaminated environment was suggested as a potential source of infection (63).

In a zoo in Missouri, USA, gradual weight loss, rough hair coat and persistent, odorless diarrhea were observed in Barbary sheep (*Ammotragus lervia*) and mouflon (*Ovis orientalis*) kept in adjacent enclosures with a rapid disease course and early death (64). After disease confirmation by histopathological examination, complement fixation titers and fecal culture, preventive control measures were initiated and both herds were culled to decrease the infection pressure on rarer and more valuable species.

A similar control regime was implemented after confirmation by fecal culture, necropsy and histopathologic evaluation in a Jimela topi (*Damaliscus lunatus jimela*), a subspecies of the African antelope species. This animal, kept in a zoo in California, USA, presented weight loss in the presence of good appetite, abdominal distention, hypoproteinemia and intermittent soft feces (65). It was euthanized to avoid the risk of potential transmission. However, in this case infection could not be confirmed in herd mates *via* serological assays and intradermal skin tests. An enormous threat of exposure and transmission of MAP in this zoological garden was confirmed by high numbers of positive individual fecal or tissue cultures obtained from different animal species of the same zoo (66). Some of these animals were kept in common enclosures. In addition, MAP was also isolated from pond water on display. A ParaTB surveillance and management program was subsequently established for this zoo and animals were defined as infected whenever MAP was isolated from feces or tissue by culture, or AFB were identified in the lamina propria of the small intestines during *post-mortem* histology (67).

The intrinsic problems of zoological gardens with MAP and ParaTB are emphasized by a report of Dukes et al. (68) on MAP dissemination in a herd of Saiga antelopes (*Saiga tatarica*; an antelope of the plains of central Asia) and their first- and second-generation offspring with mycobacterial enteritis in two zoos in Manitoba, Canada. Severe pathologic lesions consisted of varying degrees of ileal mucosal thickening, granulomatous enteritis, enlarged mesenteric lymph nodes and hepatic granulomas. Although numerous AFB were observed by histology and in direct fecal and tissue smears, only small numbers of MAP could be cultured from tissues. The frequently expressed clinical disease in young animals indicated that besides vertical transmission from infected dams, horizontal transmission from other animals was important. Not all animals with confirmed ParaTB were serologically positive in *ante-mortem* diagnosis using agar gel immunodiffusion or complement fixation (68). Most probably this outbreak resulted from potentially inadequate pre-shipment investigations before these animals were shipped from a not further specified German zoo and moved to the Canadian zoo. In Europe, the same clinical signs and pathological findings (cachexia, enlarged mesenteric lymph nodes, corrugation of the mucosa, AFB clustered in clumps in the intestinal mucosa and mesenteric lymph nodes) had already been reported before in several animals of this species with an enzootic course of the disease in Germany (69). More recently, Orynbayev et al. (70) reported one seropositive saiga antelope in the Volga-Ural region, Kazakhstan, where they collected blood-samples from 286 free-ranging animals, indicating that ParaTB also exists in the species in the natural population.

Outbreaks of ParaTB in Mishmi takin (*Budorcas taxicolor taxicolor*; a goat antelope found in the eastern Himalaya), yak (*Bos grunniens*), European bison (*Bison bonasus*), and markhor (*Capra falconeri*; a Himalayan goat) at a wildlife park in Scotland, UK, resulted in a rigorous cull and vaccination policy (71). Highest susceptibility for ParaTB and clustered disease expression was found in Mishmi takin. The animals showed pronounced weight loss, followed by an acute deterioration (72). Interestingly, due to their different birth locations and the clustered expression of disease, neonatal infection was unlikely and adult infection with rapid development of the course of the disease was suspected in this case. Signs included self-isolation, reduced appetite, malaise and abdominal pain, indicated by arching of the back and tensing of the abdomen. Infrequent and intermittent diarrhea, occurring late in disease course, and stunted growth were reported in several other cases. *Ante-mortem* fecal culture on both, herd and individual level, as well as Ziehl-Neelsen staining of fecal smears failed to detect MAP shedding. Positive results in an indirect ELISA using an unspecified anti-ruminant conjugate (ID SCREEN Paratuberculosis Indirect; ID. Vet, Grabels, France) were obtained for animals with clinical disease and in animals that developed clinical disease subsequently, indicating the advantage of multiple diagnostic tests for zoo animal testing. At necropsy, examination revealed poor body condition, granulomatous mesenteric lymphadenopathy with focal necrosis and mineralization, lymphocytic-plasmocytic periportal hepatitis and multibacillary, granulomatous and histiocytic enteritis with expansion of the lamina propria and high numbers of AFB. Positive cultures were obtained from ileum and mesenteric lymph node samples. Confirmed MAP infection in culled wild rabbits on the site was suggested as likely source of infection between enclosures of different single-species exhibits (72).

A similar infection pattern was observed in other zoos for other species. An addax (*Addax nasomasculatus*) was tested positive in a single fecal culture, in Florida, USA. However, all attempts to confirm infection with additional *ante-mortem* tests failed (73). MAP was subsequently isolated from different tissues of this animal. In addition, MAP infection was also confirmed in roan antelopes (*Hippotragus* spp.) and a springbok (*Antidorcas marsupialis*) kept in adjacent enclosures (73, 74). A culture-positive testicle of the latter may indicate that transmission *via* semen is a possible risk in this species and should be considered in other exotic ruminants. Observations in a mixed species exhibit of exotic hoofstock at the same zoo also revealed higher prevalence of fecal and tissue culture-positive animals in nyala (*Tragelaphus angasi*) and impala antelopes (*Aepyceros melampus*), both showing hypocalcemia, than in Thomson's gazelle (*Eudorcas thomsonii*), suggesting that different types of nutrition could play a role for susceptibility to infection (75). Positive fecal cultures were also found in young animals.

Without providing a more detailed investigation, several studies used fecal culture testing of zoo animals to prove exposure to MAP and support the potential risk of infection (76, 77). Results of the study by Weber et al. (77) confirmed that MAP is frequent in small zoo ruminants and fecal cultures proved to be a better method for detecting shedders than serological investigations by means of complement fixation.

Beside the cases listed above where clinical signs and disease due to MAP were confirmed or could be deduced from the findings, other reports refer the verification of infection after suspicion by tissue sample examination, culture and molecular biological techniques (78, 79). To confirm infection, Erume et al. (79) tested a nested PCR using IS900 with 15–17 copies per MAP cell as target for the rapid detection of MAP in cattle and zoo animals which were suspected to have ParaTB. The authors concluded an improved sensitivity of detection compared to bacteriological culture and single PCR. However, as stated above analog to PCR of fecal samples, negative results may be obtained depending on sample size and time of collection, and therefore do not mean absence of MAP or ParaTB.

IS900 semi-nested and quantitative real-time PCR positive fecal samples of animals without clinical signs and fecal consistency representative for healthy animals of the respective species were documented in a German zoo with known history of ParaTB (80). In another zoo in Germany, the detection of MAP DNA was also reported in samples taken from the environment as well as in feces of snow goats (*Oreamnos americanus*) and pygmy goats (81).

Probst et al. (82) tested serum samples from different zoo animals in Germany with the Chekit M.pt ELISA using monoclonal anti-ruminant IgG conjugate (IDEXX) and reported positive or suggestive results. In a study from Belgium, Vansnick et al. (55) tested zoo animals by the HerdCheck ELISA and confirmed MAP infection during *post-mortem* examination by nested IS900 PCR in tissue samples collected from the rectum of a single culled European bison (*Bison bonasus*) and in the ileum of a single banteng (*Bibos javanicus*, a species of ox found in Southeast Asia) with a skinny body condition. The risk of interspecies transmission between co-housed animals is also reported from a zoo in Turkey. Here, seropositive animals were detected by complement fixation and HerdCheck ELISA in subclinically infected goats, kept together with seropositive and clinically diseased cervids (83).

##### Cervidae

ParaTB occurs in free-ranging and captive populations of cervids and has already been reviewed extensively (4). Deer appear to be highly susceptible to MAP infection, although strong age-related resistance against disease seemed apparent (84, 85). In addition to sporadic cases in adult or mixed-age deer, outbreaks with a more acute course of disease and rapid progression to emaciation and death can occur at a younger age (e.g., fawns and yearlings) (86). Whereas deer seem to be susceptible to both type B and S strains, cattle type strains appear to be more common and virulent than sheep type strains.

Early reports on suspicion of ParaTB in captive wild cervids have been reviewed by Katic (58). However, they describe typical pathological and histopathological findings without confirmation by culture. Bourgeois (87) reported cases of ParaTB in Sika deer (*Cervus nippon*) and a red deer (*Cervus elaphus*). The latter case showed characteristic lesions and AFB were observed in smears of the colonic mucosa. Another potential case has been reported in Ontario, Canada, in a young, hand-reared moose (*Alces alces*). The animal presented chronic weight loss, intermittent liquid feces and suspicious necropsy results. Diagnosis was based on serological findings, the presence of acid-fast organisms in feces and AFB-positive *post-mortem* impression smears of lymph nodes and intestinal contents (88). Such early reports without identification of the acid-fast bacilli (AFB) by PCR for MAP-specific genetic targets must be interpreted with caution. The same caution in interpretation of results applies to the detection of extra- and intra-cytoplasmic acid-fast organisms in the intestinal mucosa, mesenteric lymph nodes and hepatic granulomas of a roe deer (*Capreolus capreolus*) and a white-tailed deer (*Odocoileus virginianus*) in Germany (63) as well as to clinically diseased and seropositive red deer (*Cervus elaphus*) with typical histological lesions in Turkey (83).

A ParaTB-like disease caused by AFB was also documented in a Belgian zoo in a captive herd of thin and emaciated pudu (*Pudu puda*), a South American deer, with terminal diarrhea and dull hair coat in infected individuals (89). Pathological findings included enteritis with thickening of the intestinal mucosa, enlarged and caseated lymph nodes and tuberculous nodules in lung and liver. Histological lesions were infiltrated with epithelioid cells and giant cells and acid-fast organisms were found in smears of intestinal content and tissues. However, only the isolates from the mesenteric lymph node of one animal were identified as MAP, whereas strains isolated from other animals on media without mycobactin were identified as rough colony type *Mycobacterium avium*. These results exemplify that suspicious histopathological lesions, either paucibacillary or multibacillary, can be indistinguishable from other mycobacterial diseases. Lesions and histopathology assessment of ParaTB have also been described in free-ranging pudu in Chile (90), which are known to shed the bacterium in feces (confirmed by triplex nested PCR using IS900 and ISMap02 as targets) and appear to be a true animal spillover host (91). In addition, MAP was also detected by culture in fecal pellet samples of wild huemul (*Hippocamelus bisulcus*) inhabiting the same remote and supposedly pristine areas in Chilean coastal Patagonia (92). This case reveals the impact of the disease on free-ranging endangered populations, which exist in low population densities with limited contact to domestic animals.

Necrotic and mineralized lung lesions, similar to those reported for *Mycobacterium bovis* infection in other wild and domestic ruminants, were described in a single tundra reindeer (*Rangifer tarandus*) of a zoological garden in Scotland, UK. This animal showed fecal soiling, chronic weight loss and subcutaneous edema (93). Besides this unusual presentation of MAP infection, granulomatous lesions in the ileum, mesenteric lymph node and liver were consistent with typical ParaTB and MAP was confirmed by PCR using IS900 and *Hsp*65 as targets. ParaTB was also reported in reindeer and red deer with positive fecal samples, clinical signs and evidence of calcification in mesenteric lymph nodes in a wildlife park in Scotland, UK (71). Infections with MAP in deer may therefore cause significant problems, not least because of tuberculosis-like caseous lesions in affected organs and interference with diagnosis of tuberculosis.

The diagnosis of infection based on the isolation from fecal or tissue cultures or the detection of acid-fast organisms during post-mortem histology was reported for many different cervid species in a zoo with a known history of ParaTB in California, USA (66, 67). Culture-positive fecal samples have also been reported in another study (76), although such results are insensitive, do not confirm infection and therefore should not be used alone to make ultimate decisions.

Positive serological tests in exotic cervids in zoos were obtained using the HerdCheck ELISA (55, 82).

##### Giraffidae

Only two reports describe the presence of MAP in zoo giraffes (*Giraffa camelopardalis*). In one study showing the molecular diversity of MAP isolates, a strain (C5) from an unspecified sample obtained from a captive giraffe was analyzed by restriction fragment length polymorphism (RFLP) (19). A second report mentions the intensive investigation of a reticulated giraffe in Florida, USA (*Giraffa camelopardalis reticulata*), which was kept in isolation after a single positive fecal culture. However, subsequent testing revealed no further positive evidence (94).

MAP infection was also confirmed in a Belgian zoo during *post-mortem* examination in one okapi (*Okapia johnstoni*) with diagnosed colitis by nested PCR of a tissue sample of the mesenteric lymph node. The animal had developed diarrhea after transportation and was positive in one fecal culture. One out of 22 fecal samples tested positive by IS900 PCR (55). A suspicious antibody ELISA result (HerdCheck ELISA) for an okapi was reported in one out of 25 serum samples in the same study.

One explanation for the low incidence of MAP in browsing herbivorous animals, which include the giraffidae, might be that in the wild they feed predominantly or exclusively on dicotyledonous plant material, including the leaves and twigs of trees and shrubs, herbs and forbs, but also on wild fruits (95). Since the fecal-oral route is the main source of MAP transmission, browsing species specialized on foliage (leaf-eaters) could therefore be less exposed to a potential contamination of the ground.

##### Moschidae

Two serum samples of musk deer (*Moschus moschiferus*) from a German zoo reacted positive in the HerdCheck ELISA (82).

### Non-ruminant Zoo Animals

The known host range for MAP infection and disease in non-ruminant wildlife has been reviewed by Hutchings et al. (96). The data raise the possibility that non-ruminant species could play an active role in the current epidemiology of the disease in livestock and can present a significant challenge to control efforts. Identical MAP genotypes found in animal species cohabiting the same property demonstrate the possibility of interspecies transmission and wildlife reservoirs of infection (19).

The pathology of MAP infections in non-ruminants is usually subtle, suggesting that most species may be dead-end hosts for the organism and will not act as self-sustaining reservoirs of infection for sympatric animals. Macroscopic lesions are extremely rare and clinical cases with histopathological findings in the intestines are sparsely recorded. Little has been published specifically on the occurrence of clinical signs, the pathogenesis and the course of infection among these species in zoos. Hence, the significance of MAP infections in non-ruminant wildlife, either free-ranging or managed in zoos, is still unknown.

While this review focuses on reported cases in zoological gardens, cases of non-ruminant free-ranging animals are addressed in the corresponding chapters.

#### Artiodactyla

##### Camelidae

Although limited information is available regarding the epidemiology of ParaTB in camelids, several cases of infection or disease have been previously reported in wild and domestic populations of camelids (97–102). Clinical signs and pathomorphological changes are similar to those seen in cattle, which was already evident in early publications and was reviewed by Katic (58). Accordingly, this suborder occupies a special position among the non-ruminant species. Clinical signs in camelids seem to occur also in young animals and the course of disease is possibly more rapid (4). Diarrhea, weight loss and hypoproteinemia are accompanied with typical pathological lesions, that may also include lymph node necrosis and mineralization as well as multiorgan dissemination (41).

Although occurrence of ParaTB seems not to be rare or unique in tylopods, only a few reports of cases from zoos are reported. Appleby and Head (103) described a case of suspected ParaTB in a llama (*Lama glama*) with lesions in the jejunum and mesenteric lymph nodes. These features of the progressive chronic disease were also illustrated in a report of clinical signs and *post-mortem* lesions in a dromedary camel (*Camelus dromedaries*), euthanized because of presumptive ParaTB diagnosis (104). Although MAP was cultured from a fecal sample, complement fixation test and intradermal skin test with johnin and tuberculin showed negative results.

Granulomatous enteritis in llamas (105) and alpaca (*Vicugna pacos*) (106) can also be caused by *Mycobacterium avium* subspecies *avium*, hence, AFB in ParaTB-like lesions of camelids must be confirmed by further testing.

Münster et al. (107) reported an alpaca in a German zoo, which showed normal appetite and had no signs of diarrhea despite its cachectic body condition, chronic weight loss and ill thrift with dullness, poor coat and pale mucous membranes. A biopsy revealed ileocecal and mesenteric lymphadenopathy and thickened intestines. Further tests by IS900 real-time PCR and culture were positive for MAP in feces and in the extirpated ileocecal lymph node. Pooled fecal samples of Bactrian camels (*Camelus bactrianus*) and many other species were found to be positive by IS900 PCR in the same zoo of which a known clinical ParaTB history had previously been reported (80).

Mycobacterial culture isolates attributed to MAP were found in water samples and fecal samples of Bactrian camels and llamas in an Ukrainian zoo (76). Rabbits experimentally infected with these isolates developed clinical signs and intestinal lesions consistent with ParaTB, highlighting the potential threat of interspecies transmission.

Considering ParaTB diagnosis in camelids, similar to cattle, serological assays can be used for rapid presumptive diagnoses in advanced cases, i.e., when showing diarrhea and weight loss, and as a screening tool for herds known to be exposed to infection (108). Furthermore, the refinement of camelid-specific ELISAs may also be a beneficial tool (109). However, seropositive results should be confirmed by direct tests for pathogen detection.

##### Suidae

Although MAP infections have sporadically been reported in studies on free-ranging suids from Spain, Korea and the Czech Republic (110–113) or in experimentally infected domestic pigs (*Sus scofra domesticus*) (114) and MAP was found in ParaTB-like granulomatous lesions (115), clinical signs in swine are dubious. Natural transmission of MAP from a goat to a Vietnamese pot-bellied pig with progressive inappetence, lethargy, diarrhea and consistent gross pathology and histopathology was reported by Hancock et al. (116).

In a zoo setting in which ruminants also tested positive, MAP was detected by culture and IS900 PCR analysis of fecal and tissue samples from a suspicious, but unspecified “wild swine” (79).

#### Perissodactyla

##### Equidae

Clinical ParaTB or presumptive diagnosis of the disease have been previously reported in equines with typical clinical signs and pathomorphological changes with numerous histiocytes and giant cells, containing AFB (117–119). Smith (120) reported the isolation of MAP (resp. *Mycobacterium johnei*) from the mesenteric lymph node in one of 100 apparently healthy domestic horses (*Equus caballus*). Identification was based on growth characteristics and animal inoculation studies in different species (121). Experimental intravenous and oral infection in domestic horses induced weight loss and the infected foals developed gross lesions in the ileum and granulomatous lesions throughout the gastrointestinal tract, where MAP was recovered from the intestinal mucosa (122). The described lesions resemble those of idiopathic granulomatous enteritis, an equine enteric disease characterized by chronic weight loss, hypoproteinemia and granulomatous, often transmural inflammation of the intestines, for which an association with mycobacterial infections is conceivable (123).

Naturally acquired ParaTB with typical histological lesions and MAP-positive tissue cultures was diagnosed in pygmy goats and in an emaciated pygmy ass (*Equus asinus form. dom*.) in a zoo in the Netherlands with a previous history of paratuberculosis in ruminants (124). Such indications of interspecies transmission between ruminants and equines have also previously been reported in a study by Eveleth et al. (125). They found positive reactions to intradermic Johnin in horses and mules kept together with diseased ruminants.

Only one report of the detection of MAP is documented in non-domestic equines. Münster et al. (80) reported positive IS900 semi-nested PCR and real-time PCR results of pooled fecal samples from Chapman's zebras (*Equus quagga chapmani*) in a zoological garden in Germany with known ParaTB history in bovines and camelids.

##### Rhinocerotidae

A questionable diagnosis was reported by Bryant et al. (126) where MAP was cultured from one fecal sample of a Southern black rhinoceros (*Diceros bicornis minor*) caught in Zimbabwe and transported to an Australian open-range zoo. Upon translocation the animal showed progressive loss of body condition and recurrent episodes of soft, poorly formed and malodorous feces over a period of four months. The culture isolate was identified by RFLP as cattle type strain. Normalization of fecal consistency and body condition was observed after antimycobacterial therapy with rifampin and pyrazinamide and administration of an oral anti-diarrhea suspension. Follow-up cultures remained negative. A false-positive result or the passive excretion of MAP in the absence of infection seems likely in this case. Otherwise, ingestion of contaminated forage or water could potentially result in a transient infection in rhinoceroses.

Positive culture (fecal or tissue specimen, not specified) was documented in a Southern white rhinoceros (*Ceratotherium simum simum*) during a survey for MAP in a zoo in California, USA, where multiple species and contaminated pond water tested positive (66).

##### Tapiridae

Antibodies against MAP were detected in a Malayan tapir (*Tapirus indicus*) from a zoo in Belgium, by HerdCheck ELISA (55). In addition, infection was confirmed by IS900 PCR in a tissue sample of another tapir (*Tapirus sp*.) suggestive of having ParaTB (79).

#### Rodentia and Lagomorpha (Glires)

Positive MAP detection in pooled fecal samples of Desmarest's hutias (*Capromys pilorides*) and Patagonian maras (*Dolichotis patagonum*) in a zoological garden in Germany are the only reports for rodents kept at a zoo (80).

Cases of infection in free-ranging rodents have been reported in species of Cricetidae and Muridae (22, 112, 127–130), many of which appeared sympatric to diseased ruminants. In addition, numerous species of laboratory animals, including mice, rats, hamsters, guinea pigs and rabbits have been experimentally infected to test their suitability for studying ParaTB [reviewed by Begg and Whittington (131)].

Reports on free-ranging lagomorphs describe infection and histopathological lesions in European rabbits (*Oryctolagus cuniculus*) (132–136), as well as the isolation of MAP from tissues and feces of European brown hares (*Lepus europaeus*) (127, 137, 138), mountain hares (*Lepus timidus*) (129) and eastern cottontail rabbits (*Sylvilagus floridanus*) (22). The increasing evidence assessed by IS900 PCR and culture that European wild rabbits pose a wildlife reservoir for MAP and the high prevalence in several populations may contribute to the persistence of infection in sympatric animals (139).

#### Non-human Primates

The occurrence of MAP in non-human primates was sporadically reported in Callitrichidae, Cercopithecidae and Lemuridae. MAP DNA has been detected in a German zoo by semi-nested and quantitative IS900 PCR in pooled fecal samples of cotton-top tamarins (*Saguinus oedipus*), gelada baboons (*Theropithecus gelada*) and black-and-white ruffed lemurs (*Varecia variegate*) (80). MAP excretion by black-and-white ruffed lemurs was highest in comparison to other animal species examined in this study, including ruminants. No clinical signs suggestive of ParaTB were evident at the time of sampling and the fecal consistency was representative for healthy animals of the respective species. Therefore, the presence of estimated MAP genome numbers in the feces of these non-human primates provide only indirect evidence of infection, as presence can be due to mere pass-through of the ingested pathogen. Nevertheless, even shedding animals must be considered as potentially infectious.

Fechner et al. (140) assumed asymptomatic infection after detection of MAP DNA by semi-nested and real-time IS900 PCR in the ileum of a cotton-top tamarin in a German zoological garden and in the bone marrow of a common marmoset (*Callithrix jacchus*) in a German primate center. However, MAP detection by cultivation failed. Also, in this case the animals did not display indicative clinical symptoms and showed no gross pathological changes. Hence although mild, mainly plasma cellular duodenitis and ileitis and activated mesenteric lymph nodes were found in the cotton-top tamarin, the causes of death were probably unrelated to MAP infections.

To date, only few cases of infections capable of causing typical granulomatous enteritis and clinical signs in non-human primates have been described in the literature. Naturally occurring ParaTB, confirmed by culture of fecal samples and different tissues, serology and restriction endonuclease polymorphism typing of ribosomal DNA, was documented in a colony of stump-tailed macaques (*Macaca arctoides*) in a primate research center in Georgia, USA. Here, the predominant clinical signs included recurrent chronic diarrhea and progressive weight loss (141). Juvenile and adult individuals were affected by the outbreak and shed the organisms with the feces. Some animals developed clinical signs and died after an average clinical course of 5 months, from the development of clinical signs to death. Serological tests indicated a high infection rate in this animal population. However, no antibodies could be detected neihter before the onset of the disease nor during the clinical stage. Pathological and histopathological findings in individual animals consisted of mesenteric lymphadenopathy and severe granulomatous enteritis extending from the upper jejunum to the ileum. Acid-fast organisms were found in histiocytes infiltrating the lamina propria and distending into the intestinal mucosa, as well as in mesenteric lymph nodes, focal hepatic granulomas, granulomas of the renal pelvis, bone-marrow and spleen. Clinically diseased stump-tailed macaques improved upon treatment with rifabutin. Similar histologic lesions were documented in a single mandrill (*Mandrillus sphinx*) held in a zoo in Illinois, USA (142, 143). Prior to death, the animal presented recurrent watery diarrhea, a distended abdomen and progressive weight loss. Necropsy and histologic evaluation revealed a severely distended abdomen, firm and enlarged lymph nodes, as well as granulomatous inflammation throughout the intestine. The intestinal lamina propria was diffusely expanded by massive numbers of Ziehl-Neelsen positive, rod shaped bacteria, accumulating within macrophages. MAP infection was confirmed by radiometric culture and IS900 PCR. Even though the number of reported cases is still low, confirmed MAP infection and active disease in stump-tailed macaques and a mandrill reveal that Cercopithecidae are susceptible to MAP infections.

Furthermore, MAP was detected by microscopic examination or direct IS900 and IS1311 PCR in fecal samples originating from six colonies of free-living rhesus macaques (*Macaca mulatta*) in India presenting coughing and loose feces (144). The presence of MAP in fecal samples was most probably a simple pass-through phenomenon. However, genotyped as “Indian Bison type,” these results also confirm interspecies sharing between domestic livestock and non-human primates in India. An earlier report of presumptive histological lesions in rhesus monkeys could not be substantiated by culture (145). Although clinical history, gross appearances and histopathologic features were suggestive of ParaTB, the importance of molecular biological methods in diagnosis was emphasized. Other mycobacterial infections (e.g., by *Mycobacterium avium* subspecies *avium*) may cause a similar gastrointestinal disease, indistinguishable by post-mortem examination or immunohistochemistry (146).

Overall, MAP infection should be considered as a differential diagnosis in primates with intestinal disease, when they develop signs similar to those of other animal species infected with ParaTB. Indicative are chronic wasting and concomitant negative test results for other potential pathogens that affect the gastrointestinal tract.

#### Hyracoidea

##### Procaviidae

Recently, a first report described MAP occurrence and infection in captive, wild-born rock hyraxes (*Procavia capensis*) and their captive-born offspring (147). Wild-born animals showed episodes of mild irregular diarrhea, but all routine parasitological and bacteriologic tests performed were negative. MAP DNA was detected by semi-nested IS900 PCR in individual and pooled fecal samples as well as in tissue samples of the gastrointestinal tract, urogenital tract, cardiovascular system and respiratory system. Sequence analysis of the DNA amplified from fecal samples showed identity to the IS900 reference sequence of the MAP-K10 genome. No MAP-specific *post-mortem* lesions were observed by gross pathology and histology and no antibody response was detected in individual serum samples. Culture was positive only from few tissue samples of the gastrointestinal tract and no positive fecal culture was obtained. Nevertheless, this species might be a possible source of MAP infections for valuable animal stock in zoological gardens and for domestic livestock in its range.

#### Diprotodontia

##### Macropodidae

The only report of MAP detection in macropods from a zoo was documented by Münster et al. (80) in a survey of pooled fecal samples from red-necked wallabies (*Macropus rufogriseus*) in Germany. The prevalence of ParaTB in free-ranging macropods seems to be very low and it is unlikely that these animals play any significant part in the epidemiology of the disease (148). Isolates of positive radiometric cultures from ileum and the associated lymphatic tissue of Western gray kangaroos (*Macropus fuliginosus fuliginosus*) and Tammar wallabies (*Macropus eugenii decres*) on Kangaroo Island in South Australia were confirmed to belong to the sheep strain of MAP (149). Despite variable gross lesions, enlargement of the ileocecal and mesenteric lymph nodes, thickened ileum and corded mesenteric lymphatics, only one culture positive animal of each species had microscopic lesions indicative of ParaTB. Indicative lesions in other animals were presumably due to infection with other fastidious non-paratuberculosis mycobacteria. Negative fecal cultures indicated that excretion of large numbers of viable MAP is rare in macropods, but they might become infected or passively excrete MAP that survive passage through the intestinal tract. Pass-through but not active infection was also concluded in a single Eastern gray kangaroo (*Macropus giganteus*) in New South Wales, Australia, where acid-fast material had been detected in a fecal smear and radiometric culture was positive for MAP. Histopathological examination of ileum and mesenteric lymph node revealed no evidence of ParaTB (150). Nevertheless, the reported cases imply a potential risk of disease transmission by infected macropods. The same applies to the frequent detection of infection in brushtail possums (*Trichosurus vulpecula*; Family: Phalangeridae) on deer farms with a history of ParaTB in New Zealand (136).

#### Carnivora

No reports of MAP detection in carnivores from zoological gardens were found. However, studies in free-ranging animals suggest that the consumption of infected prey or carrion may turn them into passive carriers. In Scotland, UK, MAP was cultured from tissues of red foxes (*Vulpes vulpes*), stoats (*Mustela erminea*), weasels (*Mustela nivalis*) and European badgers (*Meles meles*) with acid-fast positive lesions (127, 151). Anderson et al. (152) detected MAP-specific DNA by PCR targeting IS900 and HspX in intestinal tissues of scavenging mammals in Wisconsin, USA [red fox, coyote (*Canis latrans*), raccoon (*Procyon lotor*), skunk (*Mephitis mephitis*) and feral cat (*Felis catus*)]. Viable MAP was cultured from the ileum and lymph node of one coyote.

Tissue cultures of Eurasian otters (*Lutra lutra*) with unspecific chronic lymphadenitis of the retropharyngeal and mesenteric lymph nodes and acid-fast rods in imprint-cytology were positive by IS900 and F57 PCR (153). Further investigations reported infection in more free-living species in Portugal [red foxes, European badger, Eurasian otters, beech martens (*Martes foina*) and Egyptian mongooses (*Herpestes ichneumon*)] (154). Gross pathology was only observed in mesenteric lymph nodes of foxes and mongooses, while the majority of infected animals developed no visible lesions. Kopecna et al. (155) reported MAP positive cultures from the intestinal mucosa of free-living brown bears (*Ursus arctos*) from the central European Carpathians in Slovakia. The isolates were classified as a cattle type, which had been detected in domestic ruminants in the same area. However, examination of the animals did not reveal any lesions in the gastrointestinal tract that are pathognomonic for ParaTB. Infections in previously listed species have also been described in studies by de Lisle et al. (156), Corn et al. (22), Deutz et al. (129), Palmer et al. (130), Florou et al. (128), Pedersen et al. (157), and Nugent et al. (136). Miller et al. (158) reported positive culture results and clinical disease in a domestic dog in South Africa with enlarged mesenteric lymph nodes and granulomatous inflammation. The absence of visible and microscopic lesions in addition to the absence of MAP detection by culture and PCR let Sobrino et al. (159) suggest that free-ranging wolves and foxes in Spain play no relevant role in the epidemiology of ParaTB, although few ELISA-positive tests on fox sera for MAP-specific antibodies indicate contact with mycobacteria.

Carnivores can therefore act as spillover hosts and scavengers appear to be most at risk of becoming exposed and infected. Feeding of high-quality eviscerated meat in zoos may be breaking the cycle of infection seen in free-ranging carnivores.

#### Other Mammalian Species

Furthermore, MAP infection in wildlife was reported in opossums (Order: Didelphimorphia; Family: Didelphidae) (22, 152) and armadillos (Order: Cingulata; Family: Dasypodidae) (22), as well as shrews (Order: Eulipotyphla; Family: Soricidae) (22, 112) and hedgehogs (Order: Eulipotyphla; Family: Erinaceidae) (136).

#### Aves

Münster et al. (80) reported a wide dissemination of MAP in a zoo in Germany. In this study, beside positive results in artiodactyls, perissodactyls, rodents, marsupials and primates, positive IS900 semi-nested PCR and real-time PCR results were obtained also from pooled fecal samples of birds [emus (*Dromaius novaehollandiae*), parrots (*Ara* spp.), snowy owls (*Bubo scandiacus*), vulturine guineafowls (*Acryllium vulturinum*)]. Another case report of an ostrich (*Struthio camelus*) kept outdoors in Florida, USA, described positive serological diagnosis with a commercially available agar gel immunodiffusion (AGID) test for MAP antibodies, and detection of acid-fast organisms in granulomatous lesions during *post*-*mortem* examination. Bacteria were isolated on Herrold's egg yolk medium with and without mycobactin and were afterwards identified as *Mycobacterium avium* (160). This case highlights once again the urgent need for a microbiological assessment in cases where a mycobacteriosis has been diagnosed and a potential MAP infection is suspected.

Although avian mycobacteriosis is mostly caused by *Mycobacterium avium* subspecies *avium* and *Mycobacterium genavense* (161), in rare cases there is also evidence for MAP infection in free-ranging and domestic birds [Anseriformes (Anatidae); Charadriiformes (Laridae, Scolopacidae); Cuculiformes (Cuculidae); Galliformes (Phasianidae); Passeriformes (Corvidae, Estrildidae Locustellidae, Passeridae)] (22, 127, 129, 136, 162, 163). In contrast to the experimental infection of young chicken, where focal granulomatous lesions with AFB could be demonstrated (164), such histopathological changes are only documented in one carrion crow (*Corvus corone*) (127).

## Perspective

As the review highlights, a relatively small number of zoos have actually published clinical cases. However, the reports demonstrate that MAP may represent an underappreciated threat to animals in zoological gardens, especially if considering the high likelihood of unrecognized cases and that cases may go unreported. Furthermore, published data should be handled with caution. An incorporation into the defined case definitions by Whittington et al. (8), Table 1, was only possible in about one third of the reported cases. Data to separate infection from disease were not available in the majority of cases. Classification was not possible due to an unimplemented or undocumented pathological and histopathological examination or confirmation of the causative agent to be MAP by PCR for specific genetic targets. For this reason, case definitions should also be considered for zoo animal cases and the informative value of diagnostic tests should be consistently assessed when dealing with the management of suspicious individuals or populations.

### Susceptibility and Epidemiology

A better understanding of the epidemiology of MAP in wildlife can help closing the knowledge gaps that hamper the prevention and control of ParaTB (165). Since distribution of MAP in wildlife is not yet clear, animals kept in zoological gardens might provide a good opportunity to assess virulence, transmission pathways, and epidemiology of MAP, as well as the susceptibility of different animal species to ParaTB and their clinical appearance.

A variety of clinical cases with associated matching pathological findings were clearly described in ruminant species and camelids. It is important to point out that not all exposed animals become infected and not all infected animals develop clinical disease.

However, MAP infection resulting in inflammatory gastrointestinal disease seems not to be restricted to ruminants (16), although ruminants represent the main reservoir. The majority of infections diagnosed by cultural and molecular analysis in non-ruminant species were asymptomatic without characteristic histopathologic lesions or matching clinical signs. In some cases, either MAP was detected in fecal samples or antibodies against MAP were detected by serological analyses, but infection or disease could not be confirmed by *post-mortem* examination of these species. These results prove exposure to the bacterium but are only suggestive of infection. Since infected animals can host and excrete the bacteria, non-ruminants might play a role in direct or indirect transmission of the pathogen. Therefore, the ability of species to carry the organism and infect a susceptible host, as well as the potential risk of contracting a disease for sympatric ruminants needs to be further evaluated. Identification of additional potential host species and transmission pathways are important to contain the unrecognized spread of MAP. In several discussions from the reported literature, likely critical disease transmission pathways in zoos are frequent movement in and out of the collection for the purposes of maintaining captive populations, as well as mixed-species exhibits exposing animals of varying susceptibility.

The general epidemiologic features and the immune response of non-domestic animals are probably similar to those studied in domestic ruminants (15, 31). Transmission seems to follow the pattern described in cattle (166). Differences in clinical and pathological manifestation of different host species seem to exist, but confirmation by additional studies is required. Consistent with studies in domesticated species, the evaluation of long-term surveillance data in zoo animals identified a likely role of perinatal dam-to-offspring transmission (intrauterine, fecal-oral, or trans-mammary) in captive ruminant species (67). Furthermore, early-life contact with infected animals in shared enclosures during the first week of life is an important predictor of infection risk, although the effect size is smaller than that described for maternal infection status (167). Therefore, both, vertical transmission from infected dam and horizontal transmission by a contaminated environment, should be evaluated and considered as source of an infection in zoos. However, to investigate the pathogenesis in zoo animal species, more information is needed on infection rates and disease in these species. It should be determined in which species and at which age an increased risk of infection exists, to which extent infectious animals excrete the pathogen and which infectious dose represents a risk of transmission.

The potential impact on health and welfare of artificially managed populations of different endangered species, ruminant and non-ruminant, should therefore be evaluated in further research. With regard to this, it is also important to identify predisposed species and to assess the situation in free-ranging populations.

### Diagnostics and Case Definitions

Because of the lack of validated tests for zoological species and the documented instances of other mycobacteria causing diseases resembling ParaTB, diagnosis of MAP infection is challenging and difficult. For the interpretation and evaluation of clinical signs and diagnostic tests in zoo animals, we therefore recommend the case definition approach of Whittington et al. (8). The diagnostic value of corresponding findings and their limitations are listed in Table 1. Table 2 lists the proposed classification criteria and recommended case definitions for zoo animal cases based on the results of diagnostic findings.

**Table 2:** Recommended case definition terminology for ParaTB in zoo animals; adapted from Whittington et al. (8).

**Case definition**	**Finding and interpretation**	
Suspicious	AFB Se	Not sufficient to define infection Not sufficient to define infection Positive outcomes on more than one occasion increase the confidence about infection, but are not definitive
Exposed	C-f or G-f History	Culture or PCR (confirmed) from feces: strong evidence for exposure, not infection Direct or indirect contact with known-infected animals or contaminated environment Positive outcomes on more than one occasion increase the confidence about infection, but are not definitive
Infected	C-t or G-t	Culture or PCR (confirmed) from tissue (*post-mortem* or biopsy)
(Potentially) Infectious	C-f or (G-f)	Positive (and confirmed) culture results in excretes (e.g., feces) or secretes (e.g., milk) Infectious dose is not accurately known for non-domestic species
Diseased	Hp + C-t ± G-t	Demonstrable histopathological lesions consistent with MAP infection, with or without gross pathology or AFB Culture or PCR (confirmed) from tissue (*post-mortem* or biopsy)
-clinical		With clinical signs consistent with ParaTB in domestic species
-subclinical		Without clinical signs consistent with ParaTB in domestic species

*AFB, Acid-fast bacteria; Se, Serology; C-f, Culture of feces; G-f, Genome in feces; C-t, Culture of tissue; G-t, Genome in tissue; Hp, Histopathology*.

Clinical signs, gross pathology and histopathology in zoo animals are, as in domesticated species, non-specific and depend on age, species, and the stage of the infection or disease. Histologic indication must be confirmed by PCR for appropriate genetic targets because AFB other than MAP may be present.

Culture methods are slow, laborious, expensive, and require experienced technicians. Cultivation is not complete until the isolate has been verified to be MAP by PCR for appropriate genetic targets. Given the vagaries of diagnostics for MAP, only a combination of MAP detection and histopathology (assuming an appropriate number and type of tissues are thoroughly examined) can be used for a definitive diagnosis.

PCR methods continue evolving and may differ considerably in DNA extraction methods (with or without magnetic beads to capture DNA), genetic target, and method of interpretation, with real-time PCR being the most common today. In general, real-time PCR methods have replaced culture-based methods in most major diagnostic laboratories. Specifying the genetic target is particularly important when citing any PCR diagnostic results (52). IS900 remains a favorite target sequence for amplification of MAP specific loci. Furthermore, the use of lesser-used genetic targets (e.g., ISMav2) should be reconsidered, as a revision is required (168). ParaTB surveillance in exotic species should therefore be based on the use of verified MAP-specific targets and would benefit from the use of multiple genetic targets for a more reliable and validated diagnosis.

In most zoo animals, ELISAs are at best screening assays of unknown but probably very low sensitivity and varying specificity. Moreover, ELISAs vary widely in their design, i.e., coating antigens that differ in composition and conjugates that differ in reactivity with immunoglobulins of different animal species. ELISAs are not sufficient to define infection. For these reasons, ELISAs should not be recommended as a sole testing strategy but may be a useful adjunct test.

The compilation of the described cases shows that in many studies negative results are obtained although there are strong indications of infection or disease. A reliable *ante-mortem* diagnosis and early detection at individual level, particularly in valuable and endangered species, should therefore be based on a combination of different diagnostic methods that should be interpreted in series rather than in parallel, in order to enhance the accuracy of disease classification. Repeated sampling ensures a more likely identification of asymptomatic shedders and allows to monitor and prevent the spread of the bacterium (166). Reliance on confirmation in direct PCR assays of fecal samples in the absence of proof of the living organism is not recommended in culling decisions, especially when endangered species are affected. Confirmed culture-positive results or the presence of other indicators of infection are needed to avoid erroneous euthanasia (1).

Another critical aspect of MAP infection is the interference with diagnosis of other mycobacterial diseases (2). Both, the manifestation of the disease and the diagnostic challenges can cause confusion with tuberculosis. A precise distinction and diagnosis are therefore of enormous importance to maintain MAP-free status, which might ease the basic requirements for the implementation of animal transports between zoos.

### Prevention and Control

Many reports suggest that fecal-oral transmission of MAP occurs in zoo animals. Transmission and infection may be intensified when fecal contamination of the environment is not reduced by strict and standard hygiene measures. Regular cleaning of indoor and outdoor enclosures improves husbandry and leads to reduced infection pressure and prevention of infection not only in young animals. Furthermore, infection of susceptible zoo animals through contaminated feed should also be considered and excluded. It has been demonstrated that ingestion of feed contaminated by wildlife feces represented a significant potential route and source of diseases such as ParaTB to livestock (169). Therefore, implementing a strong zoo-wide pest control program is advised.

Since ParaTB is spread in herds, MAP diagnostic measures should focus on herd-mates and offspring, as well as socialized ruminant and non-ruminant species and the environment. In accordance with programs applied to domesticated species, measures in zoos must aim to examine and reduce the prevalence of infections and to prevent spread to other susceptible animals. A regular and continued survey in defined routine control and prevention programs is advisable to evaluate the infection status and to remove or isolate infected animals.

The introduction of individuals with unknown infection status or of subclinically infected animals into an existing paratuberculosis-free group represents a significant risk of transmission. A risk-based animal- and institution-specific approach for pre-shipment testing and quarantine period in zoos, based on historical prevalence of transmissible diseases, using comprehensive pathology and preventive medicine data was suggested by Marinkovich et al. (170). The combined effects of surveillance programs for different infectious diseases diminish the serious risk for animals of substantial individual value and the integrity of captive endangered species conservation programs.

Guidelines for general preventive measures, animal transfers, monitoring programs for units with negative test status, initial surveillance programs for low risk or unknown status collections and control programs for infected units can be found in the proceedings of the workshop on diagnosis, prevention, and control of ParaTB in non-domestic hoofstock (171). The establishment of appropriate ParaTB Management Units (animals, species, enclosures, geographic areas, or institutions of concern) for the purpose of diagnostic testing, surveillance, control and animal movement have been proposed and might be useful in management schemes.

Further examinations and prevention measures should consider the spread of infection within and between zoological gardens and focus on existing transmission pathways to ensure and improve effective and comprehensive ParaTB surveillance and control programs. Zoo veterinarians would benefit from explicit and specific recommendations on testing frequency and sample pooling in individuals or herds using and amending published recommendations (171). These guidelines for control programs should include monitoring and management of individual animals in single-species exhibits or multiple species in one enclosure. The use of a consistent classification for the obtained results in these investigations, as applied and suggested in this review, will allow a better assessment of incidence and frequency distribution of infections, diseases and clinical cases.

## Author Contributions

MR, TK-W, AAEW, C-PC, and RG: review conception and research design. MR: data collection and analyses. SH, TK-W, WMRO, AAEW, and RG: methodological advice and contribution to the writing of the manuscript. MR and WMRO: writing of the manuscript. All authors: contributed to the article and approved the submitted version.

## Conflict of Interest

The authors declare that the research was conducted in the absence of any commercial or financial relationships that could be construed as a potential conflict of interest.
